# Omental Prolapse Through Vaginal Cuff Dehiscence

**DOI:** 10.5811/cpcem.2022.2.56353

**Published:** 2022-07-27

**Authors:** Jon Solberg, Karan Saravana

**Affiliations:** * University of North Dakota, Department of Emergency Medicine, Bismarck, North Dakota; † University of North Dakota, School of Medicine and Health Sciences, Bismarck, North Dakota

**Keywords:** vaginal cuff dehiscence, omental prolapse, vaginal mass

## Abstract

**Case Presentation:**

A 31-year-old female presented to the emergency department with abdominal pain and a 15-centimeter bloody vaginal protrusion, which resulted during an attempted bowel movement. Reduction of the mass was unsuccessful, and the patient was taken to the operating room for examination.

**Discussion:**

In patients with a history of vaginal hysterectomy, the vaginal cuff can dehisce and abdominal contents may protrude through the vaginal canal. In this case presentation, the vaginal mass was found to be omental tissue, which could be mistaken for a prolapse of vaginal mucosa. Therefore, a proper pelvic exam is imperative, as prolapse through a cuff dehiscence can lead to severe complications.

## CASE PRESENTATION

A 31-year-old female with a history of laparoscopic-assisted vaginal hysterectomy two years prior presented by ambulance to the emergency department with an acute onset of abdominal pain and a vaginal protrusion that occurred while straining to pass a bowel movement. Physical examination was notable for a flat but slightly tender abdomen, normal bowel sounds, scant vaginal bleeding, and a 15-centimeter long, blood-tinged mass protruding from the vagina.

A brief and unsuccessful attempt at reduction was made by the emergency physician. Obstetrics and gynecology was consulted, and the patient was taken to the operating room for exam under anesthesia.

## DISCUSSION

The diagnosis was omental prolapse through vaginal cuff dehiscence. Following a vaginal hysterectomy, the vaginal cuff is closed surgically.^1^ Occasionally, this site can dehisce, allowing abdominal contents such as the small bowel or omentum to enter the vagina or protrude through the vaginal canal.^2^, ^3^ In existing literature, reports of prolapses of omentum are uncommon, and photographed cases may illustrate an anterior or apical vaginal bulge, as opposed to a completely visible omental mass, as seen here.^4^ Vaginal cuff dehiscence is estimated to have a rate of 0.39%. It is more commonly seen after total laparoscopic hysterectomy (1.35%) compared with laparoscopic-assisted vaginal hysterectomy (0.28%).^4^

Risk factors include trauma from sexual intercourse, repetitive Valsalva maneuvers, smoking, malnutrition, anemia, diabetes, immunosuppression, and corticosteroid use.^2^ Cases typically present as vaginal spotting or post-coital bleeding, and occasionally as pelvic pressure or protrusion.^2^ Most cases occur within weeks to months after the procedure, but some can present years later. Patients are at risk for infection due to exposure of peritoneal contents to vaginal and skin flora. Management includes administration of broad-spectrum antibiotics. Partial dehiscence can be managed with rest, but large dehiscence is usually managed surgically.

This case highlights the importance of the pelvic exam in patients with vaginal bleeding and abdominal pain, and care should be taken to not mistake protruding omental tissue for prolapsed vaginal mucosa.

**CPC-EM Capsule**

What do we already know about this clinical entity?

*Vaginal cuff dehiscence is seen in patients with a history of vaginal hysterectomy. Abdominal contents can prolapse through the vaginal cuff and protrude through the vagina*.

What makes this presentation of disease reportable?

*The image illustrates a rare photograph of omental prolapse via cuff dehiscence*.

What is the major learning point?

*This condition will appear as a bloody mass, which should not be mistaken for prolapsed vaginal mucosa, due to risk of infection*.

How might this improve emergency medicine practice?

*Immediate recognition allows for decreased misidentification and decreased chance of risk, such as infection of peritoneal contents*.

## Figures and Tables

**Image:**
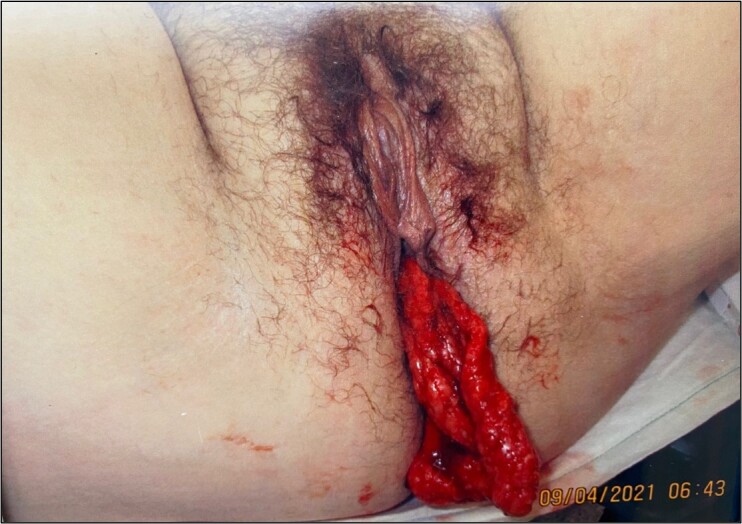
External pelvic examination demonstrates a blood-tinged mass (arrow) protruding from the vagina.
